# The Glutaminase-1 Inhibitor 968 Enhances Dihydroartemisinin-Mediated Antitumor Efficacy in Hepatocellular Carcinoma Cells

**DOI:** 10.1371/journal.pone.0166423

**Published:** 2016-11-11

**Authors:** Diancheng Wang, Gang Meng, Meihong Zheng, Yonghui Zhang, Aiping Chen, Junhua Wu, Jiwu Wei

**Affiliations:** 1 Jiangsu Key Laboratory of Molecular Medicine, School of Medicine, Nanjing University, Nanjing, 210093, China; 2 Nanjing University Hightech Institute at Suzhou, Suzhou, 215123, China; National University of Singapore, SINGAPORE

## Abstract

Reprogrammed metabolism and redox homeostasis are potential targets of cancer therapy. Our previous study demonstrated that the kidney form of glutaminase (GLS1) is highly expressed in hepatocellular carcinoma (HCC) cells and can be used as a target for effective anticancer therapy. Dihydroartemisinin (DHA) increases intracellular reactive oxygen species (ROS) levels leading to cytotoxicity in cancer cells. However, the heterogeneity of cancer cells often leads to differing responses to oxidative lesions. For instance, cancer cells with high ratio of GSH/GSSG, a critical ROS scavenger, are resistant to ROS-induced cytotoxicity. We postulate that a combinatorial strategy firstly disrupting redox homeostasis followed by DHA might yield a profound antitumor efficacy. In this study, when HCC cells were treated with a GLS1 inhibitor 968, the ROS elimination capacity was significantly reduced in HCC cells, which rendered HCC cells but not normal endothelial cells more sensitive to DHA-mediated cytotoxicity. We further confirmed that this synergistic antitumor efficacy was mediated by excessive ROS generation in HCC cells. NAC, a ROS inhibitor, partly rescued the combinatorial cytotoxic effect of 968 and DHA. Given that GLS1 is a potential antitumor target and DHA has been safely used in clinic, our findings provide new insight into liver cancer therapy targeting glutamine metabolism combined with the ROS generator DHA, which can be readily translated into cancer clinical trials.

## Introduction

Hepatocellular carcinoma (HCC) is the fifth most common malignancy worldwide and the third leading cause of cancer mortality [1]. Despite the progress in the treatment of HCC, the overall 5-year survival rate is about 12% [2]. Novel and effective therapeutics against HCC are urgently needed.

Artemisinin (Qinghaosu), a natural product of the plant Artemisia annua L, has been widely used in the past several decades as an effective antimalarial drug [3]. Dihydroartemisinin (DHA), a semisynthetic derivative of artemisinin, has been shown to have potent anticancer activity. DHA induces excessive intracellular ROS generation in cancer cells, which could be a vital shock to the cancer cells [4, 5]. Several studies have investigated the anticancer activity of DHA in lung cancer [6, 7], chronic myeloid leukemia [8], glioma [9], ovarian cancer [10, 11], leukemia [12, 13], pancreatic cancer [14–16], nasopharyngeal carcinoma [17], breast cancer [18], oral cancer [19], and hepatocellular carcinoma [20, 21]. Importantly, the safety of DHA is high, which makes it a good candidate as an anticancer agent [22].

However, while many cancer cells are under increased oxidative stress, they may benefit from relatively reducing conditions as a result of their increased intracellular ROS scavenger systems. Compared with normal cells, malignant cells survive with higher levels of endogenous oxidative stress in vitro and in vivo [23, 24]. Glutathione (GSH), a tripeptide consisting of glutamate, cysteine, and glycine, is a key member of ROS scavenging or anti-oxidative system. Cancer cells at various progressing stages may have different GSH/ROS levels. The GSH/ROS level heterogeneity of cancer cells leads to various responses to oxidative injuries [25].

Aerobic glycolysis, termed the “Warburg effect,” is a well-known phenomenon observed in most cancer cells whereby pyruvate is directly converted to lactic acid instead of entering the citric acid (TCA) cycle, regardless of the presence of a sufficient oxygen supply [26, 27]. Therefore, some critical metabolic TCA cycle intermediates are absent. To compensate these alterations and to maintain a functional TCA cycle, cancer cells often elevate glutaminolysis, by which glutamine is catalyzed by glutaminase (GLS) to glutamate and further to α-ketoglutarate (α-KG) in mitochondria. Two forms of GLS have been identified in humans and are encoded by two genes, GLS1 and GLS2 [27]. GLS1 is the key enzyme that catalyzes glutamine to glutamate and ammonia in cancer cells.

In addition to its critical role in reprogrammed glutaminolysis, GLS is also indispensable for enabling cancer cells to adapt to oxidative stress [28]. GLS1 not only controls the availability of glutamate necessary for *de novo* GSH synthesis [24, 29], but it also provides a source of reducing equivalents (NADPH, reduced nicotinamide adenine dinucleotide phosphate) required for maintaining GSH in its reduced state [30, 31]. Therefore, targeting GLS1 could result in both the inhibition of glutaminolysis and reduction of the amount of the key ROS scavenger GSH [27].

The small compound 968 has recently been found to inhibit oncogenic transformation by targeting mitochondrial GLS1 [32, 33]. Our previous study demonstrated that GLS1 is highly expressed in HCC and can be used as a target for effective anticancer therapy [27, 34].

We postulate that a novel therapeutic strategy combining GLS1 targeting with ROS induction may yield better antitumor efficacy for HCC. In this study, we investigated how 968 sensitized HCC cells to ROS-induced cell death by DHA.

## Materials and Methods

### Cell culture

Human HCC cell lines and human ECV304 endothelial cells were obtained from the Chinese Academy of Sciences Cell Bank of Type Culture Collection (CBTCCCAS). All cells were propagated in Dulbecco’s Modified Eagle Medium (DMEM) supplemented with 5% fetal bovine serum, 2 mM L-glutamine, 100 U/L penicillin, and 0.1 mg/mL streptomycin (all from Life Technology, Grand Island, NY) and maintained in a humidified incubator with 5% CO_2_ at 37°C.

### Antibodies

The antibodies for glyceraldehyde-3-phosphate dehydrogenase (GAPDH, MB001, 1:5,000 dilution), GLS (ab93434, 1:1,000 dilution), Caspase-3 (#9662, 1:1,000 dilution), and PARP p85 fragment (G734A, 1:1,000 dilution) were obtained from Bioworld (Minneapolis, MN), Abcam (Hong Kong, China), Cell Signaling Technology, and Promega (Madison, WI), respectively.

### Reagents

DHA was purchased from ShangHai Pure One Biotechnology (#P0286). The compound 968 was obtained from Calbiochem (#352010). Working solutions were prepared by dissolving the compound in dimethyl sulphoxide (DMSO) before the experiments. The final concentration of DMSO was < 1% in all experiments. Trypan blue (#T6146) and N-acetylcysteine (NAC) were obtained from Sigma-Aldrich (Saint Louis, MO, USA).

### Cell viability assays

The number of viable cells was determined by trypan blue exclusion assays. Cells were harvested by trypsin/EDTA (Life Technology, Grand Island, NY) and then stained with 0.4% trypan blue staining solution for 5 minutes.

In some experiments, cell viability was assessed by crystal violet staining assay. Cells were seeded in 12-well plates overnight followed by various treatment. Then supernatant was discarded and cells were stained with 1% gentian violet dye (diluted with methanol) solution for 30 min. Then cell plates were washed with water and naturally air-dried prior to scanning.

### MTT assay

Cells were seeded into 96-well plates and treated with the indicated compounds. Cell viability was determined after 48 h of incubation by adding 20 μL of MTT (5 mg/mL). After slightly aspirating the MTT-containing medium after 4 h of incubation, 100 μL of isopropanol was added to solubilize the formazan, followed by shaking for 15 min. The absorbance at 570 nm was recorded using the Thermo Scientific Microplate Reader (VERSA max).

### Western blot analysis

Cells were lysed in RIPA buffer containing a protease inhibitor cocktail (Roche, Mannheim, Germany, 11873580001), and the protein concentration was determined. Proteins were separated by sodium dodecyl sulfate-polyacrylamide gel electrophoresis (SDS-PAGE) and electrophoretically transferred onto a polyvinylidene fluoride (PVDF) membrane (Roche, 03010040001). After blocking with 5% nonfat milk in Tris-buffered saline containing 0.1% Tween-20, the membrane was incubated with specific primary antibodies, followed by incubation with appropriate horseradish peroxidase-conjugated secondary antibodies. Signals were detected using an enhanced chemiluminescence reagent (Millipore, Darmstadt, Germany, WBKLS0500) and the Alpha Innotech FluorChem-FC2 imaging system (Alpha Innotech, San Leandro, CA).

### Flow cytometry

ROS generation was quantified by the Reactive Oxygen Species Assay Kit (Beyotime Inst. Biotech, Jiangsu, China, S0033). Briefly, cells were harvested and incubated with the oxidant-sensing fluorescent probe DCFH-DA for 30 min at 37°C. Then, cells were washed twice with phosphate-buffered saline (PBS) and assessed by flow cytometry. Data were analyzed using FlowJo software (Version 7.6.5, Tree Star, Ashland, OR).

### Glutaminase activity test

Cellular glutaminase enzyme activity was analyzed using Cellular Glutaminase Activity Assay Kits (GMS50374.1, Genmed Scientifics Inc., MA) according to the manufacturer’s protocol. Briefly, cells were harvested by centrifugation at 300 g for 5 min at 4°C. Then, cells were lysed with lysis buffer on ice, and supernatant was harvested and the protein concentration was determined. Then the supernatant was incubated with reaction buffer at 37°C for 30 minutes followed by stop solution (marked as solution A). The reaction substrate, enzymatic solution, and buffer were mixed at 37°C for 3 minutes prior to incubation with solution A at 37°C for 30 min. The absorbance was monitored by a spectrophotometer at 340 nm. Enzyme activity was calculated according to the kit manufacturer’s supplied formula.

### Intracellular GSH and GSSG content measurement

The intracellular GSH/GSSG ratio was analyzed using the GSH and GSSG assay kit (Beyotime Inst. Biotech, Jiangsu, China, S0053) following the manufacturer’s instructions. Briefly, cells were harvested by centrifugation at 1,000 g for 10 min at 4°C. The cell pellet was then mixed with 30 μL of 5% metaphosphoric acid, followed by two cycles of freezing and thawing in liquid nitrogen and a 37°C water bath. The supernatant was then harvested by centrifugation for further GSH and GSSG assays. The total GSH level was determined by the DTNB-GSSG recycling assay following the manufacturer’s instructions. The supernatant was further treated with 1 M 2-vinylpyridine solution to remove reduced GSH. GSSG levels were then quantified using a GSSG assay kit. The amount of reduced GSH was obtained by subtracting the amount of GSSG from that of the total GSH.

### Statistical analysis

All data are expressed as the means ± standard deviations (SDs) of the indicated number of individual experiments. Differences between groups were determined using the two-tailed Student’s *t*-test, and *p* < 0.05 was considered significant.

## Results

### The compound 968 inhibits cell growth of HCC cells

**Fig 1:**
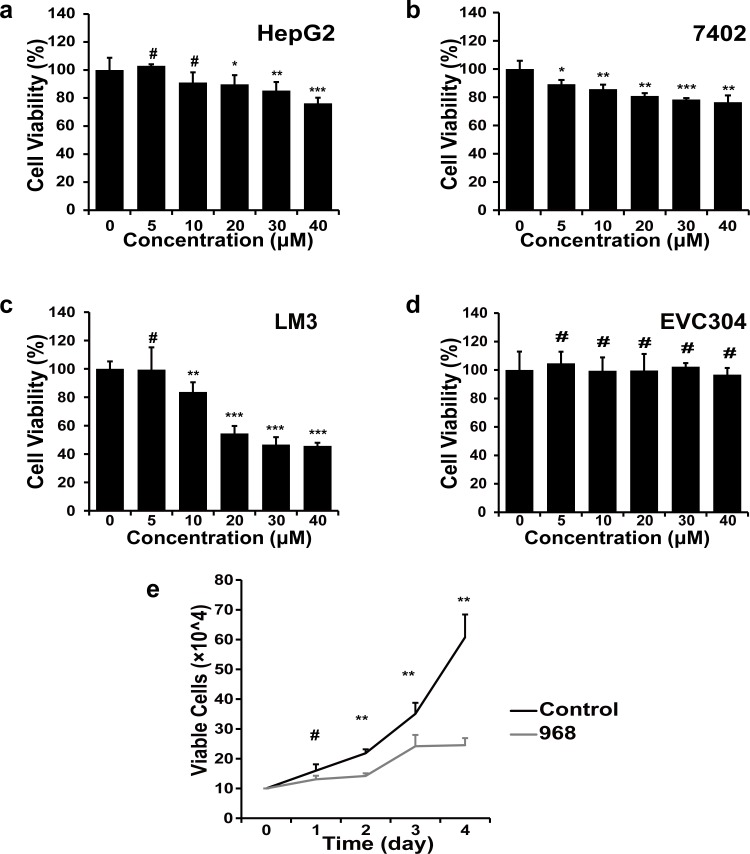
Cytotoxic effect of 968 on HCC and human endothelial cells. a-d, The inhibitory effect on cell proliferation was detected by the MTT assay after cells were treated with 968 at depicted concentrations for 48 h. Four cell lines HepG2 (a), 7402 (b), LM3 (c), and ECV304 (d) were used. Means ± SD are shown (n = 6). ^#^ not significant, *P < 0.05, **P < 0.01, and ***P < 0.001. e, LM3 cells were treated with 20 μM 968 and the viability was measured by trypan blue exclusion on 1, 2, 3 and 4, respectively. Untreated cells were used as control. Data are presented as the means ± SD of triplicates. Similar results were obtained in two independent experiments. ^#^ not significant, *P < 0.05, **P < 0.01, and ***P < 0.001.

Firstly, we evaluated the growth inhibitory efficacy of 968 in HCC cell lines. As shown in **Fig 1A–1C**, the compound 968 induced moderate growth inhibition in HepG2 and 7402 cells (**Fig 1A and 1B**) and an intensive growth inhibition in LM3 cells in a dose-dependent manner (**Fig 1C**). However, no growth inhibitory effect was observed in nonmalignant human endothelial cells EVC304, even in the presence of 968 at relative high concentrations. We also examined the dynamic viability of LM3 cells treated with 968 at 20 μM for 1, 2, 3, or 4 days, and further confirmed that 968 had a distinct inhibitory effect on LM3 cells (**Fig 1E**). Taken together, 968 inhibited HCC cells growth while showed little cytotoxicity on nonmalignant human cells.

### The compound 968 inhibits glutaminolysis leading to decreased GSH/GSSG ratio in HCC cells

**Fig 2:**
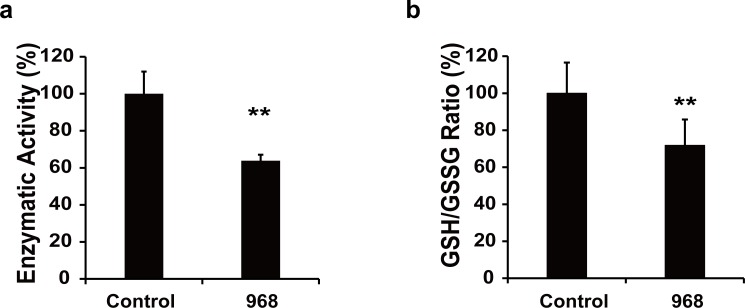
968 blocks the activity of glutaminase and decreases GSH/GSSG ratio in HCC cells. a, The activity of glutaminase in LM3 cells was tested after cells were treated with 968 at 20 μM for 24 h. Means ± SD of triplicates are shown. Similar results were obtained in two independent experiments. ** P < 0.01 compared with untreated controls. b, LM3 cells were treated with 968 at 20 μM for 36 h, then GSH/GSSG ratio was measured. The results are shown as the means ± SD of two independent experiments. ** P < 0.01 compared with untreated controls.

As glutaminolysis is crucial for cancer cell proliferation, we next examined the activity of 968 in inhibition of glutaminase in HCC cells. We found that the activity of glutaminase was decreased about 40% in LM3 cells treated with 968 at 20 μM (**Fig 2A**). In line, the inhibited glutaminolysis by 968 resulted in decreased intracellular GSH/GSSG ratio (**Fig 2B**). These data show that 968 inhibits glytaminolysis and subsequently disrupts ROS scavenger system in HCC cells.

### Cytotoxic efficacy of DHA on HCC cells

**Fig 3:**
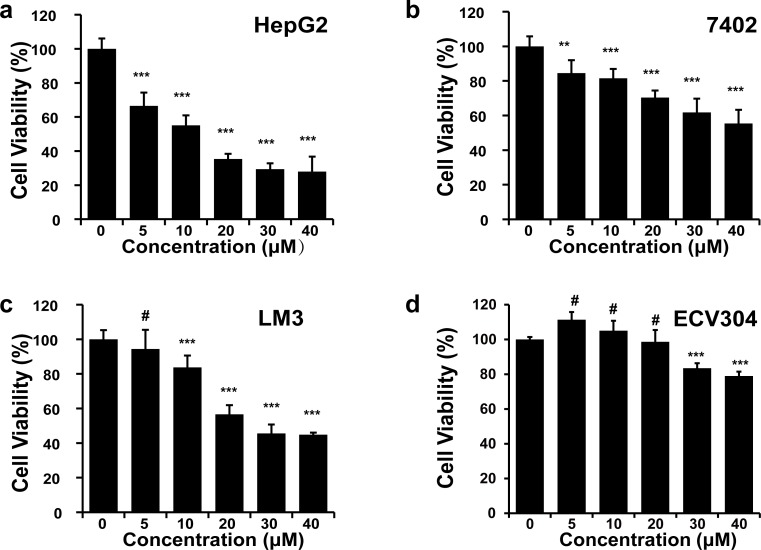
Antitumor efficacy of DHA on HCC. The cytotoxicity of DHA was assessed by MTT assay after cells (**a**-HepG2, **b**-7402, **c**-LM3, **d**-ECV304) were treated with DHA at a serial doses as indicated for 48 h. Means ± SD (n = 6) are shown. Similar results were obtained in two independent experiments. ^#^ not significant, ** P < 0.01, *** P < 0.001.

Next we evaluated the antitumor efficacy of DHA against HCC cell lines and the cytotoxicity in human endothelial cell line ECV304. We found that DHA exerted significant cytotoxicity in HCC cell lines, particularly when the concentration of DHA was over 10 μM in HepG2 cells and over 20 μM in LM3 cells (**Fig 3A–3C)**. However, EVC304 cells showed relatively resistant to DHA-induced cytotoxic effect, even at a dose of 40 μM (**Fig 3D)**. These data confirmed that DHA possesses a good antitumor efficacy against HCC.

### 968 combined with DHA exert synergistic antitumor effect in HCC cells

**Fig 4:**
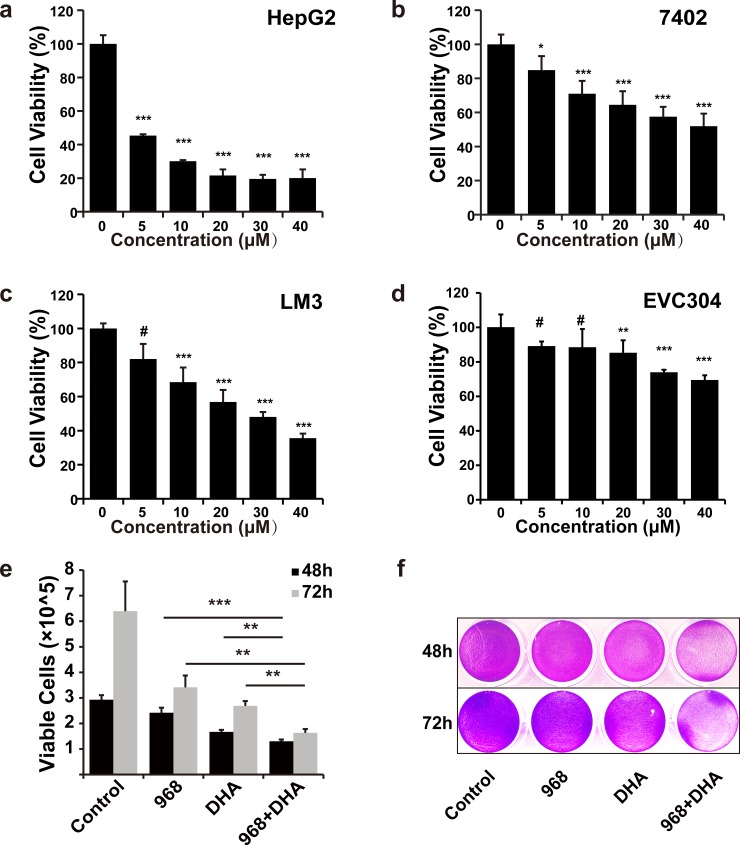
The combination of 968 with DHA exerts synergistic cytotoxic effect in HCC cells. a-d, The combinatory cytotoxicity of 968 and DHA (a-HepG2, b-7402, c-LM3, d-ECV304) was assessed by MTT assay after cells were treated with 968 and DHA at a serial of doses as indicated for 48 h. The molar ratio of 968 to DHA was 1:1. Data are presented as means ± SD (n = 6). Similar results were obtained in two independent experiments. ^#^ not significant, * P < 0.05, ** P < 0.01, and *** P < 0.001. e, The combinatory effect of 968 and DHA was assessed by trypan blue exclusion using a cell counter after LM3 cells were treated with 968 and DHA at 20 μM for 48 or 72 h. Data shown are means ± SD of triplicates. Similar results were obtained in three independent experiments. ** P < 0.01 and *** P < 0.001.f, LM3 cells were treated with 968 (20 μM) and/or DHA (20 μM) for 48 h, then cell viabilities were assessed by crystal violet staining assay. The representative plate of three independent experiments is shown.

Previous studies have shown that DHA induces ROS generation, and we showed here that 968 decreased ratio of GSH/GSSG, a ROS scavenger, we then reasoned that 968 combined with DHA might resulted in an enhanced antitumor effect in HCC cells. Indeed, our data showed that, the combined treatment of 968 and DHA markedly inhibited the viability of HCC cells (**Fig 4A–4C, 4E and 4F)**. Again, the combined therapy had much less inhibitory effect on ECV304 cells (**Fig 4D**). The combination index (CI) is a known indicator of cytotoxic synergism [35]. We further evaluated the CI of 968 and DHA in HCC cells. As illustrated in **Table 1,** 968 and DHA exerted synergistic cytotoxic effects on HCC cells. These data indicate that 968 combined with DHA achieve a synergistic antitumor effect against HCC.

**Table 1:** The CI values of various concentrations of 968 and DHA in HCC cells.

Concentration of 968 (μM)	Concentration of DHA (μM)	CI value		
		HepG2	7402	LM3
5	5	0.40336	0.94763	0.39162
10	10	0.50607	0.58690	0.52322
20	20	0.63053	0.83728	0.83006
30	30	0.42769	0.91730	0.90559
40	40	0.56054	0.96910	0.82971

HCC cells were treated with a serial of combined doses of 968 and DHA, the combination index (CI) was evaluated according to the calculation methods of Chou.

A CI < 1 represents synergy, and a CI > 1 represents antagonism [35].

### 968 and DHA cooperatively induce excessive intracellular ROS resulting in profound apoptosis in LM3 cells

**Fig 5:**
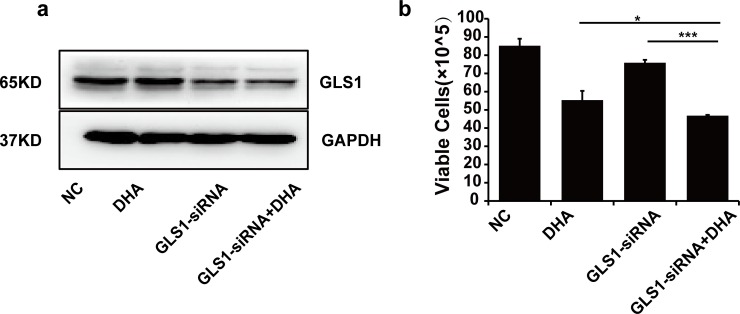
GLS1 silencing enhances DHA-induced cytotoxicity in LM3 cells. **a**, The expression of GLS1 protein was decreased after cells were transfected with GLS1-siRNA for 48 h. Blots are representative of three independent experiments. **b**, The combinatory cytotoxicity of GLS-siRNA and DHA was assessed by the MTT assay after LM3 cells were treated with GLS-siRNA and/or DHA (20 μM) for 48 h. Means ± SD are shown. Similar results were obtained in three independent experiments. * P < 0.05, *** P < 0.001.

To confirm if the synergistic cytotoxic effect of 968 and DHA is due to the specific inhibition of glutaminolysis, GLS1 was silenced by small interfering RNA in HCC cells followed by DHA (**Fig 5A**)**.** We found that specific downregulation of GLS1 enhanced DHA-mediated cytotoxicity in HCC cells (**Fig 5B**).

**Fig 6:**
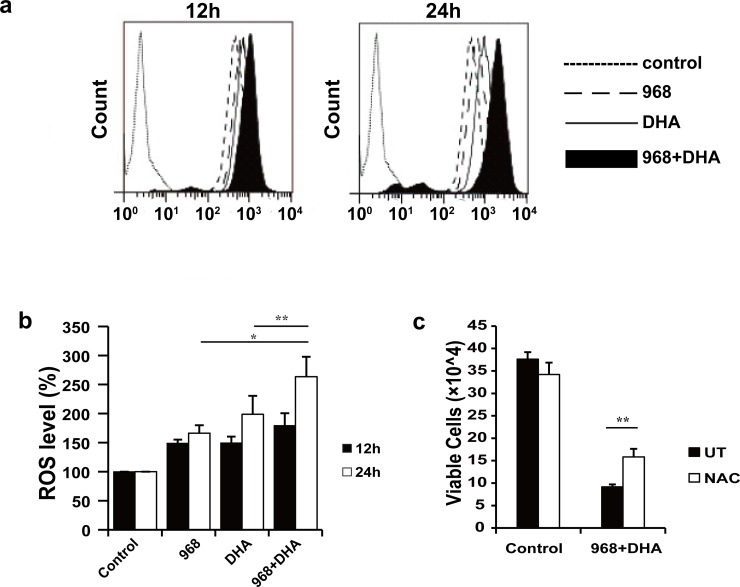
968 and DHA cooperatively increase intracellular ROS leading to enhanced cytotoxicity in LM3 cells. **a** & **b,** The intracellular ROS level was assessed after cells were treated with 968 and/or DHA for 12 or 24 h. The results are shown as the means ± SD of three independent experiments. *P < 0.05 and **P < 0.01 compared with the intracellular ROS level in cells treated with 968 or DHA only. Peak values associated with treatment for 12 h (left) and 24 h (right) are representative of three independent experiments. **c,** Cells were pretreated with NAC (3 mM) for 2 h and then treated with 968 and DHA for 48 h. The results are shown as the means ± SD of three independent experiments. ** P < 0.01 compared with cells without NAC pretreatment (UT group).

Next we wanted to know whether the synergistic antitumor effect was contributed by at least partially by increase intracellular level of ROS in HCC cells. To this end, we measured the intracellular ROS levels in LM3 cells treated with 968, DHA, or both (**Fig 6A and 6B**). We found that the intracellular ROS level was increased about 50% in cells treated with either 968 or DHA alone, and about 80% in cells treated with both for 12 h. A more distinct increase of intracellular ROS (up to 165%) was observed at 24 h in LM3 cells co-treated with 968 and DHA, compared to single treatment by 968 (up to 66%) or DHA (up to 99%). These data suggest that 968 and DHA synergistically increased the intracellular ROS levels in HCC cells.

We then further examined whether NAC, an inhibitor of ROS, could inhibit the synergistic cytotoxicity of 968 and DHA in LM3 cells. As illustrated in **Fig 6C**, NAC significantly inhibited the cytotoxicity induced by 968 and DHA, which further confirmed that the synergy was partially contributed by excessive intracellular ROS generation in HCC cells.

### Caspase-mediated apoptosis by 968 and DHA in HCC cells

**Fig 7:**
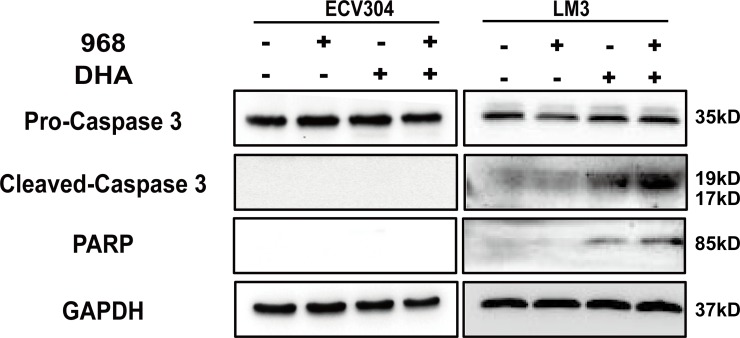
968 and DHA enhance caspase-mediated apoptosis in HCC cells. Protein expression was detected after cells were treated with 968 (20 μM) and/or DHA (20 μM) for 48 h. Blots are representative of three independent experiments.

Excessive intracellular ROS levels can induce apoptosis in cancer cells [24, 36]. We then detected PARP-1 and Caspase-3 by western blot analysis. As shown in **Fig 7**, the co-treatment of 968and DHA caused more intensive cleavage of PARP and Caspase-3 than either single treatment of 968 or DHA. Interestingly, no observed cleavage of PARP or Caspase-3 was detected in ECV304 cells. These findings indicate that enhanced apoptosis contributes to the synergistic antitumor effect of 968 and DHA in HCC cells.

## Discussion

Heterogeneous ROS scavenging capacity of cancer cells results in different responses to ROS-induced cytotoxicity. In this study, the GLS1 inhibitor 968 reduced GSH and thus sensitized liver cancer cells to ROS-induced cytotoxicity. The combination of 968 with DHA possesses several advantages in HCC therapy: 1) inhibition of altered glutaminolysis of HCC, 2) increase of antitumor efficacy while sparing normal cells, 3) potential dosage reduction of DHA. Our findings provide a novel therapeutic strategy for HCC.

Increased glutaminolysis dependence and oxidative stress are two characteristics of cancer cells, and these two characteristics could be utilized to develop new anticancer agents [24]. Due to the toxicity of glutamine mimetics, the compound 968 was then developed as an allosteric inhibitor of glutaminase, which have been intensively investigated as anticancer therapeutics targeting glutamine metabolism [31–33, 37]. The compound 968 suppresses GLS1 activity and inhibits proliferation of cancer cells [32, 33]. Our previous studies demonstrate that a kidney type of glutaminase (GLS1) is highly expressed in HCC [34], and glutaminase-specific silencing by siRNA severely disturbs ROS scavenging systems [34]. GLS1 can therefore be used as a good target for HCC therapy [27]. However, little is known about the therapeutic effect of 968 in HCC. In this study, we showed that 968 not only suppressed glutaminolysis but also disturbed the antioxidative balance in HCC cells. In addition to 968, several other compounds such as BPTES and its analog CB-839, have been shown potent antitumor efficacy via GLS1 inhibition [38–42]. Moreover, CB-839 is now being investigated in clinical trials (NCT02071862, NCT02071888 and NCT02071927). Thus, our combinatorial strategy may shift from 968 to TNBP or CB-839 for HCC treatment, which deserves further intensive investigation.

The widely accepted qualitative measure of the degree of drug reciprocity is the CI value [35]. In this study, CI was used to evaluate the combinatory effects of 968 and DHA on the toxicity of HCC cells. The compound 968 potentiated DHA-induced cytotoxicity, as revealed by CI values and the significant enhancement of toxicity in combined treatment group. The combination of 968 with DHA massively increased the intracellular ROS generation, which was synergistically caused by ROS-inducer DHA and 968, the destructor of ROS scavenging system. The excessive ROS generation in HCC cells contributed partially to the enhanced antitumor efficacy since the ROS inhibitor NAC partially abrogated cell death. We found that the synergism was various in different HCC cell lines, this was probably due to heterogeneity of ROS eliminating capability in different HCC cell lines. Interestingly, we found that lower dose of 968 and DHA combination achieved the same antitumor effect that achieved by high dose of DHA alone. This would be clinical relevant, since 968 destroys ROS eliminating capacity leading to reduced dose of DHA. Of note, the synergistic effect was not observed in normal ECV304 endothelial cells. This was probably due to relative insensitivity to ROS-mediated injury of normal cells compared to cancer cells.

In conclusion, we show here a synergistic treatment approach that targeting glutaminolysis and redox homeostasis can generate profound antitumor efficacy in HCC. The GLS1 inhibitor 968 not only blocks reprogrammed metabolism of HCC cells, but also sensitizes ROS-induced cell death via disrupting redox homeostasis, and thus might reduce the dose of DHA to a more clinical relevant level. Our study proposes a new therapeutic strategy for liver cancer that could be readily translated into clinical trials.
